# Diagnosis Accuracy of Carpal Tunnel Syndrome in Diabetic Neuropathy

**DOI:** 10.3390/medicina56060279

**Published:** 2020-06-05

**Authors:** Nicu Cătălin Drăghici, Maria Magdalena Tămaș, Daniel Corneliu Leucuța, Tudor Dimitrie Lupescu, Ștefan Strilciuc, Simona Rednic, Dafin Fior Mureșanu

**Affiliations:** 1 Centre of Advanced Research Studies, IMOGEN Institute, 400012 Cluj-Napoca, Romania; nicu.draghici@umfcluj.ro; 2 Centre for Neurological Research and Diagnostic, RoNeuro Institute, 400364 Cluj-Napoca, Romania; tudordimitrie.lupescu@gmail.com (T.D.L.); stefan.strilciuc@ssnn.ro (Ș.S.); 3 Department of Clinical Neurosciences, “Iuliu Hatieganu” University of Medicine and Pharmacy, 400012 Cluj-Napoca, Romania; 4 Department of Rheumatology, “Iuliu Hatieganu” University of Medicine and Pharmacy, 400012 Cluj-Napoca, Romania; mm_tamas@yahoo.com (M.M.T.); srednic.umfcluj@gmail.com (S.R.); 5 Department of Medical Informatics and Biostatistics, “Iuliu Hatieganu” University of Medicine and Pharmacy, 400349 Cluj-Napoca, Romania; dleucuta@umfcluj.ro; 6 Neurology Department, “Prof Dr Agrippa Ionescu” Emergency Clinical Hospital, 011356 Bucharest, Romania

**Keywords:** carpal tunnel syndrome, diabetic neuropathy, median nerve, ultrasonography, electromyography

## Abstract

*Background and objectives:* Carpal tunnel syndrome (CTS) is a common pathology, but sometimes the diagnosis is delayed in patients with diabetic neuropathy (DN). The aim of the study is twofold: first, to compare the accuracy of ultrasound (US) with that of electroneurography (ENG) in the diagnosis of CTS associated with DN, using the clinical diagnosis as a reference standard, and second, to investigate the correlation between morphological US parameters and electrodiagnosis (EDX) measurements in patients with CTS and DN. *Materials and Methods:* This study included patients with DN. They were divided into two groups: Control (patients without CTS) and Cases (patients with CTS). We performed US and ENG in both hands, totaling 56 wrists, with 28 wrists in each group. *Results:* We found that the difference in the sensory distal latencies between the median and the ulnar nerves (ring finger) exhibited the highest diagnostic accuracy of all the US and ENG parameters, areas under the receiver operating characteristic (AUC) = 0.99 (95% CI 0.97–1), and it was significantly different from the best US diagnostic method. The wrist cross-sectional area (CSA) had the most accurate US diagnosis, while the wrist-to-forearm ratio had the worst AUC. Moreover, in the group of CTS and DN patients, the wrist CSA enlargement was statistically directly proportional to the median compound muscle action potential (CMAP) distal latency and inversely proportional to the antidromic median nerve conduction study (NCS) and the orthodromic median palm–wrist NCS. *Conclusions:* Both examinations can be used with confidence in the diagnosis of CTS overlapping with DN, but the EDX examination seems to be more accurate. Furthermore, we found a positive correlation between the US and EDX parameters.

## 1. Introduction

Carpal tunnel syndrome (CTS) is the most common entrapment neuropathy [1]. With a prevalence of approximately 5% in the general population, it may be caused by various diseases and conditions which increase the pressure on the median nerve at the level of the wrist [2]. Diabetic neuropathy (DN) is one of the most common complications of diabetes mellitus (DM), with a high prevalence between 23% and 54% [3,4]. In diabetic patients, the prevalence of CTS varies between 14% in subjects without DN and up to 30% in patients with DN [5].

Ultrasound (US) examinations are non-invasive, fast, and cost-effective. They can identify structural abnormalities and categorize various causes of nerve compression. Electrodiagnostic (EDX) studies are sometimes uncomfortable, do not inform on the anatomy of the nerve, but are considered the gold standard in the clinical confirmation of CTS [6]. Contrariwise, in patients with clinically manifested CTS, some authors have found normal values of nerve conduction velocities (NCVs) but abnormal echo graphic parameters [7]. Moreover, previous studies have found that some patients with clinical CTS may have a negative nerve conduction study (NCS) [8]. Overall, the contribution of the two investigations is complementary, and these methods cannot be used to diagnose CTS in the absence of specific clinical symptoms [9].

Because DN symptoms can mimic CTS, the median nerve entrapment diagnosis in patients with type II DM may be recognized with difficulty and delay. Currently, there is no gold standard to diagnose CTS in DN. The hypothesis of the study was the median nerve US and electroneurography (ENG) are useful in the diagnosis of CTS in DN subjects.

The study aimed to compare the diagnostic accuracy of US with that of electroneurography (ENG) in the diagnosis of CTS associated with DN using the clinical diagnosis as a reference standard. Second, we wanted to investigate the correlations between morphological US parameters and EDX measurements in patients with CTS and DN.

## 2. Materials and Methods

### 2.1. Patients

We included twenty-eight consecutive adult patients with type II diabetes mellitus, who arrived in the diabetes outpatient clinic with symmetrical clinical symptoms, suggestive of a length-dependent DN.

DN was diagnosed using the following clinical criteria: (a) numbness and tingling in the lower limbs; (b) reduced or absent knee and ankle reflexes, and (c) alterations in the vibration and position sense of the hallux [10]. All cases of DN were confirmed by NCS [11]. The presence of any other causes of non-diabetic polyneuropathy, such as chronic alcohol consumption, chemotherapy, folate and vitamin B12 deficiency, were excluded. The DN subjects were divided into two groups: fourteen patients with bilateral clinical symptoms of CTS (cases) and fourteen patients without any signs or symptoms of CTS (controls).

The clinical diagnosis of CTS was established by the presence of numbness and/or tingling in at least two of the digits I, II, III, and ½ of IV persisting for at least 1 month which was aggravated during sleep, sustained hand positioning, or repetitive actions of the hand [12,13]. Moreover, the patients’ histories aimed to exclude other neuromuscular diseases, such as (a) cervical radiculopathy and/or brachial plexus neuropathy, (b) sensory symptoms of ulnar neuropathy, and (c) prior CTS surgery [13]. Patients with hypothyroidism were also excluded.

Two blinded and independent physicians performed the US and ENG measurements in both groups for all the 56 wrists. Clinical examination and ENG were performed on the same patients on the same day. The US evaluation was performed on the same subject on the same day or the next day. All subjects gave their informed consent for inclusion before they participated in the study. The study was conducted in accordance with the Declaration of Helsinki, and the protocol was approved by the Ethics Committee of the University of Medicine and Pharmacy Cluj Napoca on 9 February 2016 (Registration No. 26).

### 2.2. Neurophysiologic Tests:

All examinations were performed by a neurophysiologist with 4 years of experience using a CareFusion Viking machine. During the investigations, the skin temperature of the participant was maintained above 32 °C.

The DN diagnosis was confirmed by reduction in the sensory nerve action potential (SNAP) amplitude of both sural nerves less than 10 uV with/without decreasing the following values: superficial peroneal nerve SNAP amplitude <10 uV, distal peroneal nerve compound muscle action potential (CMAP) < 2.6 mV, distal tibial nerve CMAP < 5 mV. The sum of the SNAP amplitudes of the sensory nerves was calculated to quantify the severity of DN.

The diagnosis of CTS was established using standardized techniques for the bilateral median and ulnar nerves. The median CMAP was recorded using a cutaneous electrode (E1) placed over the abductor pollicis brevis muscle. Median nerve stimulation was performed between the palmaris longus muscle tendon and the tendon of the flexor carpi radialis muscle, 7 cm proximal from the E1 electrode. The median SNAP was recorded antidromically over the digit II, and 13 cm was measured between the stimulation and recording site. Moreover, we register an orthodromic median SNAP by stimulating the nerve in the palm, 8 cm distal to the recording electrode.

The median–ulnar motor latency difference to intrinsics (lumbrical II, respectively, interossei II muscles) were registered in the distal third of the area delimited by the second and the third metacarpal bones. The simulation was performed at the wrist, 10 cm proximal from the recording electrode. The median–ulnar sensory latency difference to the ring finger was calculated by stimulating both nerves at the wrist at the standard points. The recording electrode was fixed on the IV finger at a distance of 14 cm from the stimulation electrode [14].

We established the EDX diagnosis of CTS based on the recommendations and criteria of the American Association of Neuromuscular and Electrodiagnostic Medicine (AANEM) [13]. A median motor nerve distal latency ≥4.2 and/or an antidromic median sensory NCV < 40 ms were considered diagnostic for CTS. Likewise, a difference >0.5 ms between the median and ulnar motor nerve distal latency and/or a difference >0.5 ms between median and ulnar sensory latency were considered positive for CTS.

### 2.3. Ultrasonography

The US examination was performed using a Siemens Acuson S 2000 machine (Siemens Medical Solutions, Ann Arbor, MI, USA) and a linear 18 MHz transducer (Siemens Medical Solutions, Ann Arbor, MI, USA). An experienced rheumatologist with 9 years of practice was blinded for the EMG results and performed all examinations. The intraobserver intraclass correlation coefficient (ICC) for wrist measurements was 0.961 (95% CI 0.915–0.983), and for forearm measurements, ICC was 0.945 (95% CI 0.881–0.975). The US evaluation was performed on the same subject on the same day or the next day after the ENG examination. The upper limb was maintained in a neutral position to eliminate possible nerve deformities, and the transducer was held perpendicular to the median nerve. A large amount of gel was applied to minimize the force applied by the probe.

**Figure 1:**
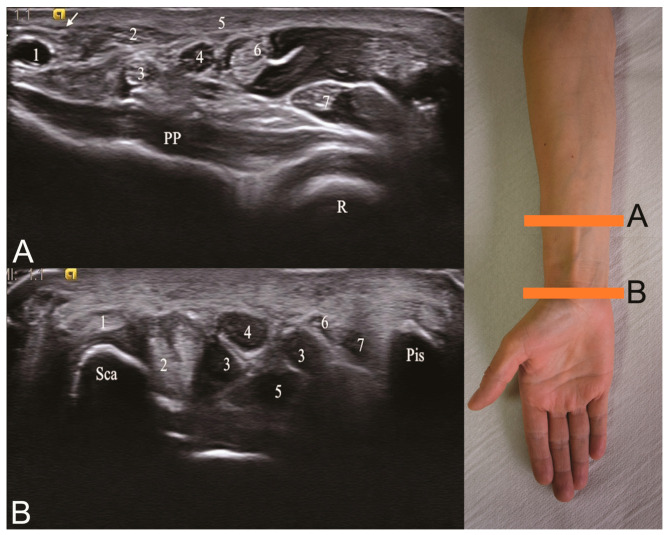
The median nerve ultrasound in the transverse plane at two different sites. The cross-sectional area (CSA) of the median nerve was measured at the wrist and 5 cm proximal to this level. (**A**) R—radius bone, PP—pronator quadratus muscle, (1) radial artery, (2) flexor carpi radialis tendon, (3) flexor pollicis longus tendon, (4) median nerve, (5) palmaris longus tendon, (6) flexor digitorum superficialis tendon, (7) flexor digitorum profundus tendon, arrow—flexor retinaculum. (**B**) Sca—scaphoid bone, (1) flexor carpi radialis tendon, (2) flexor pollicis longus tendon, (3) flexor digitorum superficialis tendon, (4) median nerve, (5) flexor digitorum profundus tendon, (6) ulnar artery, (7) ulnar nerve, Pis—Pisiform bone.

The median nerve was spotted in the transverse plane, and the cross-sectional area (CSA) was calculated by tracing the nerve two times at the wrist above the flexor retinaculum and 5 cm proximal to this level (Figure 1). At the wrist, the pisiform bone was used as a landmark throughout the investigation. A cross-sectional area of the median nerve at the wrist >12 mm² or wrist–forearm difference (WFD) > 4 mm² was suggestive of CTS [15]. The WFD parameter was calculated by subtracting the measurements obtained at mid-forearm from the wrist CSA determination. Moreover, we calculate the wrist–forearm ratio (WFR), which represents the fraction between the CSA value at the wrist divided by the CSA measurements at the mid-forearm.

### 2.4. Statistical Analyses

The categorical data were described by counts and percentages. The continuous data that exhibited normal distributions were described as mean and standard deviations, and continuous data that were not normally distributed were described as median and interquartile ranges (IQRs). The associations between categorical variables were assessed with the Chi-square test. The comparisons between the two groups regarding normally distributed variables were made using independent samples *t*-tests, while those regarding non-normally distributed variables were made using Wilcoxon rank-sum tests. The comparisons between the two groups (with and without CTS) regarding ENG parameters and US measurements were made using Wilcoxon rank-sum tests for clustered data [16].

The areas under the receiver operating characteristic (AUC) and their confidence intervals were computed nonparametric clustered receiver operating characteristic (ROC) methods [17]. The comparisons between two AUCs were performed with DeLong’s test for two paired ROCs.

The correlations between the US and ENG parameters were assessed with partial Spearman correlation coefficients, adjusted for age and diabetes duration.

For all statistical tests, a two-tailed *p*-value was computed, and a 0.05 level of significance was used. All statistical analyses were performed in the R environment (R Foundation for Statistical Computing, Vienna, Austria) for statistical computing and graphics, version 3.4.1 [18], the ICC was computed with ‘irr’ package [19], and the Wilcoxon rank-based test for clustered data was computed with clusrank package [16].

## 3. Results

**Figure 2:**
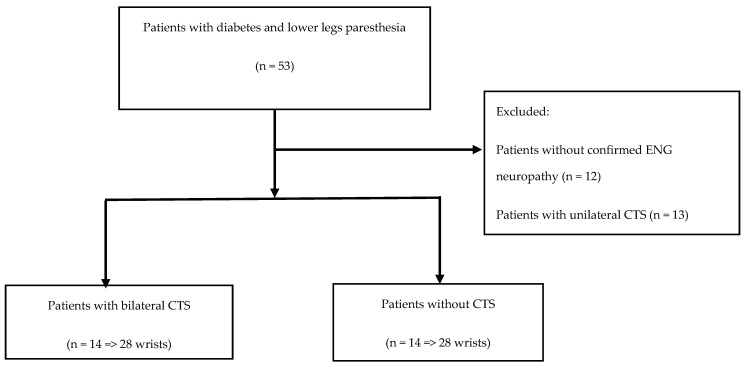
Patients flow diagram. CTS—carpal tunnel syndrome; ENG—electroneurography.

We recruited 53 patients with diabetes and length depended neuropathy symptoms, referred from diabetes physicians. All patients underwent an ENG examination. Patients without confirmed ENG neuropathy and patients with unilateral CTS were excluded. We ended up with two groups of 14 patients each, one with bilateral CTS and the other without CTS (Figure 2).

### 3.1. Patient Characteristics, US, and ENG Measurements

The comparison of the characteristics of both groups of patients, namely Cases (patients with DN and CTS) and Controls (patients with DN without CTS), is presented in Table 1. There were no significant differences between the two groups in regard to age, sex, height, weight, waist circumference, glycated hemoglobin, duration of diabetes, or diabetic neuropathy.

In Table 2, the US and EDX parameters were compared in the CTS and DN subjects versus the Controls group, and we found a statistically significant difference between the two groups. The CSA of the wrist was increased, and, at the same time, the WFR and the WFD were higher in the Cases group. On the other hand, all the EDX parameters were significantly different between the two groups; the median CMAP distal latency was prolonged in the CTS cases, and the median sensory NCV was diminished. Moreover, the difference between the median motor distal latency and ulnar motor distal latency (lumbricals II—interossei II), as well as the difference between the median and ulnar sensory distal latencies (digit IV), was also prolonged in the Cases group. The severity of DN was similar between the two groups, and there was no difference between the summated SNAPs.

### 3.2. Analysis and Correlations of US and ENG Parameters

In Table 3, we calculated the area under the curve for the US and ENG parameters using the clinical diagnosis of CTS as the gold standard.

We found that the difference in the sensory distal latencies between the median vs. the ulnar nerves (digit IV) achieved the highest diagnostic accuracy of all the US and ENG parameters. The wrist CSA had the highest US diagnostic accuracy, while the wrist–forearm ratio had the lowest. The difference in the motor distal latencies between the median and the ulnar nerves (lumbrical II vs. interossei II) had the lowest diagnostic accuracy of all the ENG parameters, but it was similar to the best of the US parameters, namely the wrist CSA.

**Figure 3:**
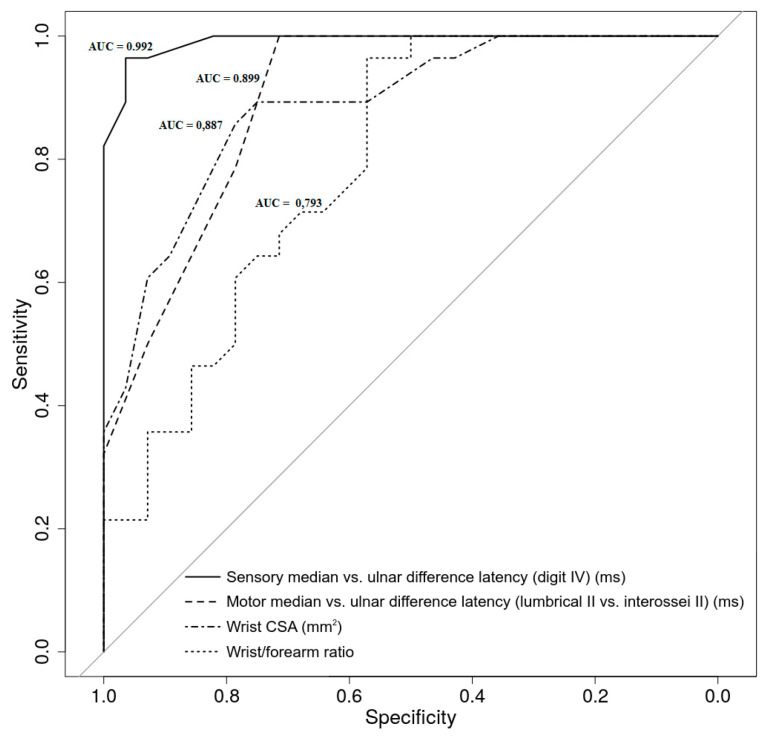
Receiver operating characteristic curves for the ultrasonographic and electrodiagnostic parameters in patients with/without carpal tunnel syndrome and diabetic polyneuropathy.

In addition, DeLong’s test was used for comparing two correlated ROC curves for the following: (a) the best US AUC (Wrist CSA) with the best ENG AUC (Sensory median vs. ulnar difference latency—digit IV) (*p* = 0.011); (b) the best US AUC (Wrist CSA) with the worst US AUC (Wrist–forearm ratio) (*p* = 0.117); and (c) the best ENG AUC (Sensory median vs. ulnar difference latency) with the worst ENG AUC (Motor median vs. ulnar difference latency) (*p* = 0.017) (Figure 3).

In the Cases group, we demonstrated the partial correlation between the US and ENG parameters adjusted according to age and duration of diabetes (Table 4).

We found a statistically significant directly proportional correlation between wrist CSA enlargement and prolonged median CMAP distal latency and an inverse proportional correlation between an increase in wrist CSA and a decrease in the antidromic median NCS and orthodromic median palm–wrist NCS.

## 4. Discussion

This study was conducted to test the hypothesis that the median nerve US and ENG are useful in the diagnosis of CTS in DN subjects. In our study, using this method, we discovered a significant difference between the two groups of patients. However, we found the EDX method to have the greatest accuracy—the difference in the sensory distal latencies between the median and ulnar nerves (digit IV) being significantly better compared to the best US method: the wrist CSA. In addition, the worst EDX method was similar to the best US method.

Regarding the CSA of the median nerve at the wrist, the WFR and the WFD, these measurements were higher in patients with CTS and DN than in the control group. Likewise, other similar studies have shown that the CSA of the median nerve at the wrist was elevated in patients with DN and was correlated with EDX parameters [20].

In other studies, authors compared two groups of patients with DN, with and without CTS. In contrast to our study, they did not find a significant difference in the CSA at the wrist or in the WFR and WFD between the DN patients with or without CTS [21]. The results could be different because there are some differences between the two studies. First, the patient enrolment methods were different, and second, the methodology for the quantification of DN severity was also different.

Hobson and his collaborators investigated the relationship between the median nerve area at the wrist and the WFR in patients with and without CTS in the general population. The author reported that the WFR of the median nerve in patients with CTS might be superior in CTS diagnosis compared to the median CSA measurement at the wrist [22]. In our group of diabetic patients, the WFR was not more accurate than the CSA at the wrist in the diagnosis of CTS, perhaps due to the diffuse enlargement of the peripheral nerves observed in patients with DM. This statement was demonstrated by a study in which the author excluded all the patients with CTS and demonstrated that the CSA of the median nerve at the wrist in DN patients was enlarged compared to patients without DN [23]. Therefore, some authors argue the idea that the diffuse increase in the CSA of the median nerve without WFR changes may be useful in the diagnosis of DN [20]. Moreover, recent studies have demonstrated that the peripheral nerve CSA was greater in patients with type II DM, especially in patients with DN [24].

Regarding the CTS pathophysiology, two components are important in our cases: the compressive and metabolic ones. These changes are the consequence of diverse mechanisms represented by (a) increased pressure in the carpal tunnel, (b) median nerve connective tissue compression and nerve tethering, (c) median nerve microcirculation injury, and (d) synovial tissue hypertrophy [25]. The mechanical factors described above can be more pronounced in diabetic patients due to microvascular changes in nerve structure, which has, as a consequence, a reduction in the endoneurial blood flow, manifested through edema, hypoxia, and neuritis [26]. Moreover, various structural changes related to the synovial tissue hypertrophy are also aggravated in diabetes by non-enzymatic glycosylation of collagen and alteration of its turnover [27]. In addition, a study on the diabetic and non-diabetic population with CTS shows that median nerve injury in diabetic patients can be aggravated by several factors, represented by hyperglycemia and neurotrophic factor deficiency [28] associated with a high tendency to develop CTS due to fragility of peripheral nerves in diabetes [29]. As a consequence, in our population, the median nerve neuropathy may be related to a mechanical factor, associated with a metabolic component, which also represents a risk factor for the compression mechanism. In our study, we did not use positive provocative tests (Tinel sign and Phalen test) for the clinical diagnosis, since some studies show that these tests lack specificity [13].

Diagnosing CTS in diabetic patients and surgical treatment in certain cases, show an increase in the long-term quality of life [30]. Moreover, conservative treatment, such as physiotherapy based on neurodynamic techniques, had a positive effect in CTS subjects with an improvement in the NCV and maintaining amelioration until 6 months after the last session of procedures [31,32]. This important observation can probably be easily applied with success in clinical practice in diabetic patients. In addition, we believe that increasing the number of diagnostic methods may increase the number of treated patients. Therefore, using the US and EDX methods, we can improve the diagnostic accuracy of CTS in patients with DN. Our paper has several limitations. The small sample size is a flaw in the study. Moreover, we did not measured the elasticity of the nerves, and, during US measurements, the mid-forearm position was not standardized and was determined in relation to the wrist position, using the pisiform bone as a landmark. Future similar studies should evaluate a greater number of patients, grouped based on the severity of the disease, to enhance the diagnosis of CTS in patients with DN.

## 5. Conclusions

In our study, both examinations were shown to be useful in the diagnosis of CTS, but the EDX examinations were more accurate. Therefore, in the diagnosis of CTS associated with DN, the most accurate method we found was the EDX examination—the difference in the sensory distal latencies between the median and the ulnar nerves (digit IV), which was significantly different from the best US method, namely the wrist CSA. Moreover, the worst EDX method was similar to the best US method.

The CSA of the median nerve at the wrist in patients with DN was higher in the control group and was correlated with NCS.

## Figures and Tables

**Table 1:** Patient characteristics between both groups.

	Cases (DN and CTS) n = 14 Patients	Controls (DN without CTS) n = 14 Patients	P
Age (years), mean (SD) (range)	62.71 (6.37) (5–74)	64.14 (6.2) (50–74)	0.55 ***
Sex (male:female)	7:7	5:9	0.44 **
Weight (kg), median (IQR)	89 (83.25–107.5)	89.5 (82–93.75)	0.74 *
Height (cm), median (IQR)	1.65 (1.6–1.75)	1.7 (1.62–1.8)	0.31 *
Waist circumference (cm), median (IQR)	115 (110–125.25)	108 (98.25–116)	0.27 *
1- Hb A1c (%), mean (SD)	7.73 (1.28)	7.36 (1.44)	0.48 ***
Diabetes duration (months), median (IQR)	126 (111–177)	138 (111–153)	1 *
Symptoms of DN duration (months), median (IQR)	30 (12–48)	24 (9.75–36)	0.54 *

CTS—carpal tunnel syndrome, DN—diabetic neuropathy, SD—standard deviation, IQR—interquartile range. Wilcoxon rank-sum test * Pearson’s Chi-square test ** independent samples *t*-test ***.

**Table 2:** Ultrasonographic and electroneurographic measurements.

	Cases (DN and CTS) n = 28 Wrist	Controls (DN without CTS) n = 28 Wrist	*p*
Wrist CSA (mm²), median (IQR)	11.5 (10.88–14.12)	9 (7.5–10)	<0.001 *
Mid-forearm CSA (mm²), median (IQR)	7.25 (6.5–9)	7 (6–7.55)	0.182 *
Wrist–forearm ratio (WFR), median (IQR)	1.64 (1.37–2)	1.3 (1.18–1.43)	0.001 *
Wrist–forearm difference (mm²), median (IQR)	4.5 (3.38–5.58)	2 (1–3)	<0.001 *
Median CMAP latency (ms), median (IQR)	4.8 (4.1–5.12)	3.5 (3.4–3.73)	<0.001 *
Antidromic median NCS (digit II) (ms), median (IQR)	34 (32.75–39.5)	48 (44–52)	<0.001 *
Motor median vs. ulnar difference (lumbrical II vs. interossei II) (ms), median (IQR)	0.85 (0.5–1.22)	0.25 (0.1–0.4)	<0.001 *
Sensory median vs. ulnar difference (digit IV)—(ms), median (IQR)	1.4 (1.1–2)	0.25 (0.1–0.4)	<0.001 *
Ulnar CMAP amplitude (ms), median (IQR)	9.45 (7.97–11.27)	10.8 (9.25–12.25)	0.27 *
Ulnar CMAP latency (ms), median (IQR)	2.7 (2.48–2.9)	2.7 (2.58–2.9)	0.69 *
Summated SNAP (uV), median (IQR)	11.5 (0–23)	9.5 (0–22)	0.31 *

CTS—carpal tunnel syndrome, CSA—cross-sectional area, SNAP—Sensory nerve action potential. SD—standard deviation, IQR—interquartile range, Wilcoxon rank-sum test for clustered data *.

**Table 3:** Comparison of the areas under the receiver operating characteristic curves between the ultrasonographic and electroneurographic measurements.

Characteristic	AUC (95% CI)
Wrist CSA (mm²)	0.887 (0.799–0.976)
Wrist–forearm ratio	0.793 (0.670–0.915)
Wrist–forearm difference (mm²)	0.881 (0.797–0.965)
Median CMAP latency (ms)	0.95 (0.895–1)
Antidromic median NCS (digit II) (ms)	0.973 (0.939–1)
Motor median vs. ulnar difference latency (lumbrical II vs. interossei II) (ms)	0.899 (0.812–0.986)
Sensory median vs. ulnar difference latency (digit IV) (ms)	0.992 (0.977–1)

CSA—cross-sectional area, CMAP—compound motor unit action potential, NCS—nerve conduction study.

**Table 4:** Partial correlation between the ultrasonographic and electroneurographic parameters in patients with carpal tunnel syndrome and diabetic neuropathy.

	CMAP Distal Latency	Antidromic Median NCS	Orthodromic Median Palm–Wrist NCS
Wrist CSA	0.747 *	−0.595 *	−0.753 *

CSA—cross-sectional area, NCS—nerve conduction study, CMAP—compound motor unit action potential, Spearman partial correlation coefficient *, *p*-value < 0.001. The values were adjusted for age and diabetes duration.
